# Defining ready for discharge from sub-acute care: a qualitative exploration from multiple stakeholder perspectives

**DOI:** 10.1186/s12913-023-09285-y

**Published:** 2023-05-02

**Authors:** Kate Gledhill, Tracey K Bucknall, Natasha A Lannin, Lisa Hanna

**Affiliations:** 1 School of Health and Social Development, Deakin University, Geelong, Australia; 2 School of Primary and Allied Healthcare, Monash University, Frankston, Australia; 3 School of Nursing and Midwifery, Deakin University, Melbourne, Australia; 4 Department of Neuroscience, Central Clinical School, Monash University, Melbourne, Australia; 5 Alfred Health, Melbourne, Australia; 6 Centre for Quality and Patient Safety Research, Institute for Health Transformation, Deakin University, Melbourne, Australia; 7 Institute of Health Transformation, Deakin University, Geelong, Australia; 8 Department of Occupational Therapy, School of Primary Health and Allied Care, Faculty of Medicine, Nursing and Health Sciences, Monash University, Peninsula Campus, Frankston, VIC 3199 Australia

**Keywords:** Clinical decision-making, Patient readmission, Length of stay, Patient discharge, Qualitative, Sub-acute Care

## Abstract

**Background:**

Planning discharges from subacute care facilities is becoming increasingly complex due to an ageing population and a high demand on services. The use of non-standardised assessments to determine a patient’s readiness for discharge places a heavy reliance on a clinician’s judgement which can be influenced by system pressures, past experiences and team dynamics. The current literature focusses heavily on discharge-readiness from clinicians’ perspectives and in the acute care setting. This paper aimed to explore the perceptions of discharge-readiness from the perspectives of key stakeholders in subacute care: inpatients, family members, clinicians and managers.

**Methods:**

A qualitative descriptive study was conducted, exploring the views of inpatients (n = 16), family members (n = 16), clinicians (n = 17) and managers (n = 12). Participants with cognitive deficits and those who did not speak English were excluded from this study. Semi-structured interviews and focus groups were conducted and audio-recorded. Following transcription, inductive thematic analysis was completed.

**Results:**

Participants identified that there are both patient-related and environmental factors that influence discharge-readiness. Patient-related factors discussed included continence, functional mobility, cognition, pain and medication management skills. Environmental factors centred around the discharge (home) environment, and were suggested to include a safe physical environment alongside a robust social environment which was suggested to assist to fill any gaps in functional capabilities (i.e. patient-related factors).

**Conclusions:**

These findings make a unique contribution to the literature by providing a thorough exploration of determining discharge-readiness as a combined narrative from the perspectives from key stakeholders. Findings from this qualitative study identified key personal and environmental factors influencing patients’ discharge-readiness, which may allow health services to streamline the determination of discharge-readiness from subacute care. Understanding how these factors might be assessed within a discharge pathway warrants further attention.

## Background

An ageing population and an increase in co-morbidities have led to an increased demand on public hospitals [1], moving non-essential care, once offered to inpatients while in hospital, to the community [2]. Effective discharge from hospital has been shown to increase quality of life for patients as well as preventing costly hospital readmissions [3]. Discharge planning pathways currently use both standardized and non-standardized assessments to assist with decision-making, with clinicians more frequently reverting to those without psychometric testing [4]. Without standardized assessments and processes to support decision-making about discharge readiness, there is a greater reliance on individual clinician judgement.

Much of the qualitative research published on discharge planning focuses on clinicians’ perspectives [5–7] rather than considering patients’ and other stakeholders’ views. Factors shown to influence clinician decision-making has included individual factors such as clinical experience and mentorship as well as clinicians’ emotional responses to past experiences, and on organizational factors such as system pressures and team dynamics [8–10]. Understanding factors that influence discharge readiness from perspectives beyond those of the clinician is currently limited. There is also little published on discharge readiness from subacute care facilities, i.e. care provided after an acute care episode, focused heavily on rehabilitating functional activities and less on stabilizing medical conditions [11].

A gap in the literature exists, with a systematic review concluding that of the 23 articles reviewed on discharge readiness from subacute care, only 1 was within a mixed-diagnostic population with the majority being in a neurological population [12]. To better understand the concept of discharge readiness from subacute care facilities, this study explored perceptions of being ready for discharge from the perspectives of key stakeholders: inpatients, family members, clinicians and managers. Given the gap in the literature, this study aimed to provide an exploration of discharge readiness from multiple stakeholders within multiple diagnostic groups. We sought to better understand the determinants of being ready for discharge in order to streamline discharge planning processes within subacute care.

## Methods

### Ethics

Institutional ethical approval was obtained prior to commencement (581 − 15) and all participants provided written, informed consent.

### Study design

This study used a qualitative descriptive design [13].

### Research team

The research team consisted of two occupational therapists (one a research student and one an experienced clinical researcher), one senior nurse (with a joint academic position and expertise in qualitative research) and one public health academic with extensive expertise in qualitative research.

### Setting

A metropolitan subacute hospital in Melbourne, Australia, was the setting for this study. This hospital has 200 beds (76 rehabilitation; 123 geriatric evaluation and management (GEM)). These 200 beds are spread across four aged care wards (general medicine), three rehabilitation wards (admitting neurological, spinal, orthopedic, trauma, general medicine, burns and amputee patients), and a dedicated acquired brain injury (ABI) unit. The average length of stay for subacute care across this facility ranged from 20 to 25 days. The GEM unit traditionally offered a slower stream of rehabilitation with a focus on discharge planning and an older patient cohort whereas the rehabilitation beds offered faster stream rehabilitation. Patients were recruited from both units, with the majority (n = 13) coming from the rehabilitation units.

### Participants

Participants consisted of four separate groups: inpatients, family members, hospital managers, and clinicians.

### Sampling and recruitment

A purposive maximum variation sample [14] of participants was selected to represent a broad range of diagnoses, experience and education. Table 1 outlines the recruitment strategy and inclusion/exclusion criteria for each participant group. Patients were recruited from a number of different wards to ensure variability in diagnostic groups.

Recruitment rates varied between stakeholder groups; all of the approached inpatients consented to participate (n = 16); however, only 16 of the 20 family members that were approached agreed to participate. Reasons for non-consent were: limitations on participants’ time (n = 1), unable to find a mutually agreeable time (n = 1), and two family members reported they wanted to focus on the inpatient’s recovery. Some dyads (n = 5) were recruited at the participants’ request, consisting of inpatients (n = 5) and family members (n = 5). Patients were approached via the lead author, who was explicit about her clinical role within the hospital; clearly explaining the lead author had no relationship with other staff on that ward. Approaching patients on weekends and after hours, outside of clinical treatment and visiting times assisted in recruitment rates.

All managers (n = 12) who were approached consented to participate, and all clinicians at the site were invited to participate with 17 consenting. Managers included department heads from nursing, medical and allied health.

**Table 1:** Recruitment of participants

Stakeholder	Recruitment
Inpatient	Admitted inpatients to the subacute setting at the time of recruitment were screened via medical records to determine eligibility. Patients who were admitted to multiple wards were approached to ensure a broad range of diagnostic groups and ages were included. Patients with a known cognitive deficit, limited English proficiency or under the direct clinical care of the lead author were excluded. Eligible participants were approached in person by the lead author and written consent obtained.
Family members	Family members were identified through recruited inpatients and approached either in person or via a telephone call. Additionally, family members of patients admitted to the aged care and neurological wards were approached via a telephone call from the lead author to capture family members of excluded patients. Family members from these wards were screened for exclusion criteria by the lead author.
Clinicians	All clinicians working within the subacute setting were approached via emails and provided verbal information at staff meetings by the lead author. Recruitment emails were sent via the Director of Allied Health to minimise feelings of coercion. Verbal information was also provided by the lead researcher at staff meetings with contact information being provided to allow clinicians to volunteer for the project.
Managers	All managers at the site were sent an email by the lead researcher and invited to participate.

### Data collection

**Fig. 1:**
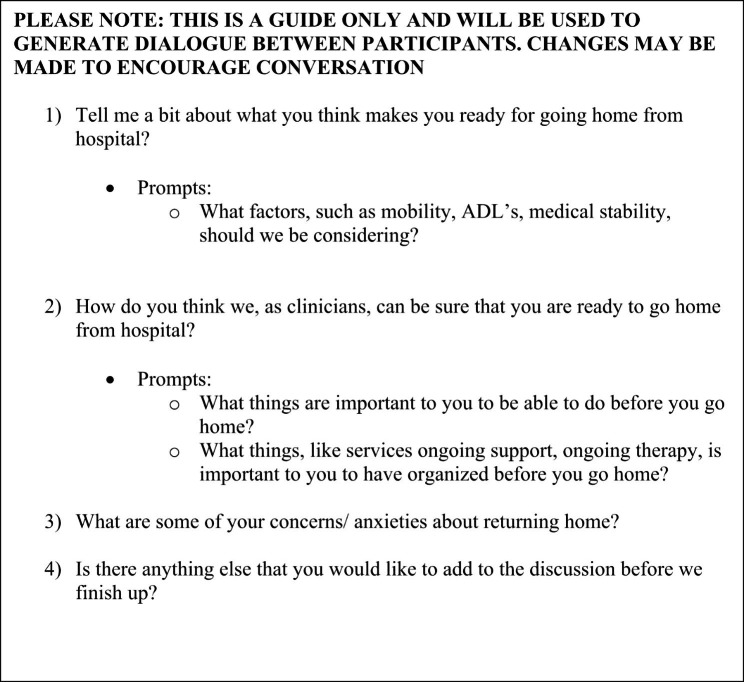
Example of Interview guide

Data were collected from all participant groups using semi-structured interviews (n = 36) [15] and/or focus group discussions (n = 6) based on participant preference [16]. Interview guides (refer to Fig. 1: Example of interview guide) were developed and pilot tested [17] on the respective participant groups before data collection commenced. Pilot-testing the managers’ interview guide was not possible given the small pool of eligible participants but was reviewed by the research team for clarity.

Inpatient interviews were conducted within the hospital in a quiet room away from other inpatients. Family member interviews were conducted either in the hospital (n = 12) or in the family member’s home (n = 4). All clinician and manager interviews and focus groups were conducted within the hospital. Field notes [18] were taken during the focus groups, with analytical memos being written immediately following each interview and focus group by the interviewer to ensure reflexivity.

Data collection was conducted over a time period of three years, allowing for data analysis to begin between interviews and focus groups, which allowed the team to build upon each interview using data from previous interviews and to determine when data saturation had occurred. No repeat interviews or focus groups were conducted. Data was collected between November 2016 to June 2019. This data was collected prior to the Covid-19 pandemic and is representative of the health service prior to the increased demand from Covid-19. Throughout the data collection period, no changes to policies or procedures in discharge planning occurred on the wards.

### Data analysis

All interviews and focus groups were audio-recorded and transcribed verbatim. Inductive analysis [19] was completed. Data were coded in a systematic process, manually without the use of software, allowing statements regarding discharge planning and key discharge criteria to be identified. Codes were sorted into sub-categories and from these sub-categories, categories [20] were determined. Two authors [KG, LH] collaborated to conduct this preliminary analysis. The same two authors [KG, LH] developed an analytical framework and this was refined by all authors through discussion, modifying and reaching a consensus on themes over multiple meetings.

Following data transcription, a one-page summary was sent to all participants from that interview or focus group, for the opportunity for member checking (16); no changes or clarification of categories were requested. Additionally, after data analysis was completed, a summary of combined themes was sent to all participants via email.

## Results

There were 61 participants in total across all stakeholder groups. Inpatients (n = 16) were aged between 29 and 83 (median age 61.9 years) and had a range of diagnoses. Participants included amputee (n = 2), spinal (n = 2), orthopaedic (n = 4) and general medicine (n = 7) patients. All family members (n = 16) interviewed were direct family members and varied in ages, educational levels and cultural backgrounds. Family members were either spouses (n = 11) of the inpatient or the inpatient’s children (n = 5). Clinicians (n = 17) ranged in experience from being new graduates (within two years of graduating) to senior clinicians; they were of varied disciplines, representing speech pathology, nutrition, social work, occupational therapy, physiotherapy and nursing, as outlined in Table 2: Participant characteristics. Managers (n = 12) were from diverse backgrounds: occupational therapists, physiotherapists, medical and nursing staff. The semi-structured interviews (n = 36) took an average of 48 min, and the focus groups (n = 6) ran for an average of 64 min. Focus groups were predominantly carried out within the clinician stakeholder group (n = 4), with one focus group consisting of patients and one with managers. Clinician focus groups consisted of four (n = 4), four (n = 4), six (n = 6) and three (n = 3) participants in each group. There were three (n = 3) participants in the patients focus group and five (n = 5) in the managers focus group.

**Table 2:** Participant characteristics

Participant group	Age range	Sex	Diagnosis/relationship to patient/Qualification of professional
Patient (n = 16)	29–83 (median 61.9 years)	Male- 31% Female- 69%	Amputee 12.5% Spinal 12.5% Orthopedic 31% General medicine 44%
Family member n = 16		Male- 37% Female- 63%	Spouse or partner 69% Child 31%
Clinicians n = 17	Less than 2 years experience- n = 2 More than 2 years experience n = 15	Male- 6% Female 94%	Medicine 6% Nursing 6% Speech pathology 12% Occupational Therapy 52% Physiotherapy 12% Social work 6% Dietitic 6%
Managers n = 12		Male 42% Female 58%	Medical 33% Nursing 42% Occupational Therapy 17% Physiotherapy 8%

Analysis of stakeholders’ accounts identified two key themes, patient-related factors and environmental factors, that influenced a patient’s readiness for discharge to minimize the level of risk for complications or readmissions. Patient-related factors included categories of personal capacity and medical status, and environmental factors incorporated categories of social and physical environment.

### Patient-related factors

This theme is comprised of two categories that contributed to being ‘discharge-ready’ from subacute care: personal capacity and medical status.

#### Personal capacity

A patient’s personal capacity for functional ability (referring to activities of daily living) and physical health included three sub-categories: continence, functional mobility (mobilizing household distances, getting on and off the toilet and in and out of bed) and cognition. Participants recognized that these three factors interrelate and impact on each other, but perceived continence as the most important consideration for discharge from hospital.

#### Continence

All participant groups spoke of managing continence or having someone that could assist with continence as essential for discharge, recognizing that continence could contribute to other health issues such as falls and skin infections.

*“I absolutely think my number one thing I think we need to focus on in hospital is continence. I think if we worked on continence, so many other things would change. There would be less falls. We know that because people often fall when they are feeling uncomfortable and vulnerable from a continence perspective.“*

*Manager 007*

Current funding structures of formal services did not provide a service that assisted with the unpredictable nature of toileting, which impacted when a patient was ready for discharge.

*“I think the toileting is something that I see as a priority. Obviously showering and dressing is also important and attending to hygiene. But I think if someone has a sponge wash on every second day and PCAs (personal care assistants) are coming in, that’s – but I think about the 24/7 picture of someone needing to go to the toilet, and we’ll never have services that are going to be there to take someone to the toilet when they need.“*

*Clinician 004*

While continence was considered as an important step towards preparedness for discharge, with implications for mental health and emotional preparedness, no patients identified it as an associated risk to their physical health. They reported, however, that necessary activities of daily living, such as toileting, were things that could make them feel normal again and to feel empowered.

*“I think you need to be able to toilet yourself and do the usual things that make you feel -there’s a certain dignity that comes from being able to clean yourself and toilet yourself. And you feel like those things keep you and you can sort of I think, get into a depressed state.“*

*Patient 008*

All family members raised continence as a source of stress for them, identifying that assisting with continence was a burden that significantly impacted their ability to care for the patient at home.

*“He wasn’t ready to go to the toilet by himself and empty the bags. It was cleaning up, you know, half a dozen times a day. It was a - just the physical in and out, in and out, in and out, on-call, this was too stressful.“*

*Family member 003*

#### Functional mobility

All participant groups identified that mobility was an important consideration in a patient being ready for discharge, but recognized that a patient’s mobility did not need to be at a pre-morbid level of function regarding gait aid and distance. Participants recognized that having assistance with mobility and transfers could mitigate these risks when returning home. For example, Patient 9 stated:

*“So, you’ve got to be able to be strong enough, and whether it’s a wheelchair or whether it’s sticks or whatever mobility you choose, you’ve still got to be able to transfer in and out.“*

*Patient 009*

Clinicians agreed with this, stating that outpatient programs implemented to continue therapy post-discharge meant that a patient needed only indoor mobility and functional transfers, getting in and out of chairs and beds and on and off the toilet, to discharge the patient safely from hospital and lessen patient length of stay:

*“The patient was able to transfer independently on the ward, walk short distances but still was probably just walking a distance from her bedroom to her lounge room. I didn’t necessarily feel it was necessary for her to stay in hospital because (community service) could go out. But if (community service) didn’t then I probably would have tried to get her to stay a little bit longer to build up her endurance.“*

*Clinician 013*

Family members agreed that patients needed to walk functional distances before being discharged and that patients requiring more assistance with functional mobility significantly increased the stress for the family member that was caring for them.

*“Maybe he should have been a bit stronger in that area (mobility). He’s really good now, because he can walk from the bedroom to the toilet, I thought if he was like that when he came home, that would have been a lot better (less stressful).“*

*Family member 003*.

#### Cognition

All clinicians and managers acknowledged that a patient’s cognitive status was a consideration for discharge-readiness; however, a poorer cognitive status was not necessarily a reason for someone not to return home. Patients and family members did not identify cognition as a consideration in a discharge planning context.

Cognitive impairment is a descriptive term used often in healthcare but all managers and clinicians in this study identified that cognitive impairment presented differently for each patient. They recognized that how a person functions was more relevant for discharge planning than one specific cognitive area.

*“I think problem-solving and planning is probably more important than just memory, per se. There’s probably some aspect that’s more important than other – rather than just overall sort of cognition domain.“*

*Manager 004*

Clinicians acknowledged that a more holistic view of the patient that considered the medical, physical, social and functional components of safety at home was required. Most clinicians, however, stated that the patient needs to have a level of awareness that is conducive to managing the support or equipment put in place and maintain safety.

*“Safety awareness in general, you know. Do they have the cognition to be able to work the equipment safely and are they making safe decisions within their home, outside their home? That’s a grey area sometimes in discharge planning.”*

*Clinician 005*

#### Medical factors

Medical factors impacting a patient’s readiness for discharge included the following subcategories: having acute medical conditions stabilized; and medication and pain management.

#### Acute medical conditions being stabilized

Managers and family members were the two participant groups that raised acute medical conditions being stabilized prior to discharge, with clinicians being the only group that spoke of chronic disease management.

All managers recognized that patients should remain in the hospital whilst they were physiologically unstable, that is while their medical conditions presented a risk to them and they needed 24-hour monitoring, intervention and care:

*“We should only use any patient bed for people who can’t be managed in the community, and that is for people who have physiological instability related to an ongoing medical problem, they’ve got high-level nursing needs that can’t be met in the community, or that there are risks that can’t otherwise be mitigated and managed. So, medical problems can be managed in the community in the home at best. It’s when that person has an instability in their medical problems which puts them at risk of potentially deteriorating rapidly.“*

*Manager 008*

However, family members were concerned about the consequences of patients being discharged before they were medically ready and identified a lack of consistency in the medical team’s determination of a patient’s medical stability, which increased their stress significantly.

*“The day he came home, he had extreme abdominal pain, and I thought, ‘Oh, this seems like a compacted bowel,‘ which it was. So, then he had to go back into the hospital, and those two nights and days he was home were a nightmare.“*

*Family member 010*.

In addition to acute medical stabilization, clinicians perceived that it was their responsibility to ensure patients were provided with the skills to manage their chronic diseases, such as diabetes, before discharge. Clinicians, however, were the only study participant group who thought chronic disease management was essential for discharge-readiness.

#### Medication and pain management

Clinicians and patients raised medication management as a key consideration for returning home. All clinicians reported that medication management was highly critical, whether the patient was independent or having someone assist and oversee this, to limit the risk of readmission.

*“It is that independent medication management, if not what services are in place to assist with med checks or whatnot because an implication from that is readmission.“*

*Clinician 005*.

All inpatients stated that knowing what they needed and being supplied with the medications required for discharge was important to them, as they thought that it was safer to have a plan that could be changed if needed, but they did not want to be left short of medications.

*“To get to see your GP you pretty much have to make an appointment three weeks in advance. So, I’d need to go from here with a plan and medication and then I’d be more than happy to throw it away if I didn’t need it.“*

*Patient 003*

Inpatients recognized that this was equally important for post-discharge pain management. They stated that they feared being stranded after discharge if their pain changed, again identifying that ongoing support at home was essential. Inpatients expressed concerns about what the plan could be if they deteriorated at home, how to seek the advice to manage any increase in pain.

*“Well, I assume at home you’re not under the guide of a clinician. I think the key is that post-discharge support. I think if there’s someone that can say, I think I’ve gone downhill or something that can flick the gears and take appropriate response to that.“*

*Patient 008*

### Environmental factors

This theme is comprised of two categories affecting a patient’s discharge-readiness from subacute care: their social environment and their physical environment.

#### Social environment

All participant groups reported that it was essential to have a strong support network in place for the patient to be discharge-ready. Formal supports were identified as those provided by external providers and informal support was that provided by the family or people from within the patient’s social network. Having family or carers available for support helped prepare patients emotionally for discharge. It significantly reduced the stress and the risk when being discharged home from both a physical and emotional perspective.

*“I wasn’t left alone, and I knew the support was going to be there, and that made me mentally feel more comfortable.“*

*Patient 006*

All family members agreed that formal support systems gave them more confidence in their own ability to cope at home, knowing that there was assistance that could reduce the level of care they needed to provide to the patient. They spoke of the impact that a robust social network mixed with informal and formal supports, had in preparing them mentally for the discharge.

*“And even to give confidence. So, giving confidence that there are resources that could come to help support me.“*

*Family member 009*

Clinicians stated that when a patient showed a reduced ability to manage functional activities of daily living, their support network and physical environment could be altered to help bridge the gap between what the patient could do and what they needed to be able to do. They recognized that having both formal and informal support at home often lessened the length of stay for patients.

*“My experience has been sometimes they don’t have anyone, and I think that really changes how long they stay in hospital. So, if someone has social supports, I think that they’re quick to be moved through.“*

*Clinician 002*

However, both patients and family members reported it was important that continued rehabilitation was arranged prior to going home from hospital. Patients felt this meant they could go home earlier, provided that the level of expertise was equal to that in hospital. There was a belief amongst some patients that hospital-based allied health staff had a greater understanding of their specific injuries than community-based clinicians.

I would like to see continuity of the people that you’ve used in here who know exactly what they’re doing and they know your history that’s important…… you see the average suburban physio I’m sure doesn’t have anywhere near the knowledge of these people in certain aspects. I’ve already asked for a full report from the physios to go to my physio but I know very well that her run of the mill sore leg from football, whatever, is a far cry from what you’ve been through and the treatments you need. That’s the concern and I’m pleased to hear, and it was only yesterday, that they will bring me back.

*Patent 001*

In contrast, family members acknowledged that having ongoing therapies arranged not only allowed for there to be improvements in the functional capacity of the patient, but also allowed family members to feel more mentally prepared for the discharge. This reassurance provided hope that the patient’s functional capacity would continue to improve, but also provided additional support and an expert to ask questions of.

*“I felt much more comfortable when I knew that there were people coming to visit every day after he came home, I knew that if there were any questions, there was someone coming in the next day that could answer them. It also meant too that someone else was looking after him as well, this was such a comfort to me”*

*Family member 016*

#### Physical environment

All participant groups agreed that having a physically safe environment to return home to was important to minimize the risk of poorer outcomes post discharge. Discussions regarding a safe environment involved both the provision of equipment or any required home modifications but also the provision of ongoing therapies. There were differences noted amongst stakeholder groups. Patients identified that their physical environment was one of the most critical factors influencing their discharge readiness, and wanted any required modifications to the home or any necessary equipment completed before discharge, as not knowing was an area of concern and anxiety.

*“I haven’t got the chair yet though, so how can they say that I can go home and fit through the doors when I haven’t got the right chair.“*

*Patient 002*

The clinicians agreed that a safe home environment was paramount, however, they stated it was acceptable to wait for some home modifications. They identified that the priority was to complete home modifications that affected the patient’s immediate safety and that all other major home modifications could wait, specifically larger bathroom modifications where the patient could potentially make alterations to their normal practice (i.e. sponge wash in place of showering) or equipment or support services could be used to minimize the risk (i.e. use of a bath board while awaiting removal of a shower over bath). The timing of home modification completion was the only factor that patients and clinicians disagreed with.

*“I often think of minimizing risk as much as possible, but I probably wouldn’t say that there are always a lot of the recommendations that are done before someone will always go home. So sometimes your kind of gap filling, minimizing the risk until that can be done in the community or until it can be sorted from a long-term point of view because you just don’t have the time to keep them in hospital.“*

*Clinician 002*

## Discussion

This study found that multiple stakeholders in subacute care (inpatients, family members, clinicians and managers) perceived that to be ready for discharge, inpatients needed to be able to maintain continence (with or without support), walk functional distances, have their acute medical conditions stabilized, take medications either independently or with support, and manage any chronic co-morbidities. In addition to these patient-related factors, having a robust social environment and a safe physical home environment was seen as paramount for discharge-readiness.

This study corroborates previous research reporting a complex interaction between environmental and patient-related factors [21]. Participants in this study identified that enhancing a patient’s physical and social environment may counteract a change or decrease in a person’s physical or cognitive functioning. The findings of this study can be significant for health services as they can provide strategies/services that may assist in streamlining hospital discharges and reduce preventable hospital readmissions.

Participants in this study identified that independence with continence or assistance to manage continence was essential for a patient to be ready for discharge home, as well as independence with functional mobility. This finding supports that of a study conducted in the United Kingdom in the acute setting, which found that patients who are incontinent of urine and need help with transfers at the time of discharge had a higher likelihood of an adverse outcome [22]. Further, studies in the United States have identified that patients in subacute care who are incontinent at discharge are more likely to be discharged to a care facility [23], which may be related to the impact incontinence has on both patients and the carers. Similarly, family members in this Australian study recognized that caring for a family member with incontinence increased their stress significantly. This finding also supports existing research in other contexts; for example, a recent systematic review identified that caring for a patient with incontinence harmed the informal carers’ physical, psychological and financial status [24]. A qualitative study conducted with people with multiple sclerosis and their carers identified the emotional toll that bowel incontinence caused for both carers and patients, identifying the need to begin discussions about bowel incontinence much earlier in order to prepare them both practically and emotionally for discharge [25]. This finding is consistent with the emphasis placed on continence management and education by all stakeholders in our study.

Research has been able to demonstrate the benefit of managing co-morbidities at time of discharge as a preventative factor in reducing readmissions [26]. These findings are consistent with our study, where clinicians perceived a strong need to address comorbid chronic health conditions prior to discharge from subacute care. Managers, however, perceived comorbidities should be managed by community health services (not perceiving this to influence discharge readiness). All stakeholders did agree, however, that the ability to manage medications prior to discharge was essential. Prior studies demonstrated that medication errors after discharge are prominent, and the impact of these are significant [27]. In an American study 12.5% of patients experienced a medication error within a month of returning home, with 62% of these being preventable [28]. Providing patients with discharge medication summaries completed by the pharmacist is one strategy that has been identified to reduce medication errors [29]. However, recent research in Australia determined errors in 61.5% of discharge medication lists in both acute and subacute care, creating a period of vulnerability for the patient in this time of transition. In addition, this same study recognized that complete medication lists were found in only 24% of discharge summaries [29]. Internationally, a study in the United Kingdom reinforced that inadequate medication explanations led to incorrect dosages and increased stress and anxiety [30]. Having a pharmacist conduct medication education before discharge may significantly reduce this risk in addition to providing an adequate handover to community medical practitioners. A randomized control trial conducted in Vietnam, in acute cardiology care, identified that patients who received pharmacist-led counselling before discharge had greater medication adherence and greater patient satisfaction [31].

A patient’s environment, both physical and social, is a crucial consideration in determining a patient’s readiness for discharge, with our study recognizing that having adequate social support resulted in patients potentially being discharged earlier and reducing the risk for readmission. This corroborates existing literature, with a study in the United Kingdom determining that older people who lived alone were at a 50% higher risk for emergency hospital admissions than those patients who lived with family [32]. Familial support was recognized as essential for discharge-readiness by participants in this current study, with all participant groups recognizing the benefit of having supports at home. A qualitative study conducted in the United States further reinforced this, finding that social isolation and a lack of support and assistance from friends and families were concerns patients had about returning home and should be recognized and assessed as a risk factor in discharge from hospital [33]. Improving the provision of services, such as assistance with daily living activities and assistance in managing medications or chronic health conditions, could help bridge the gap between hospital and home and may be one potential strategy to better outcomes post discharge. Recently, in Australia, a nurse-led telephone call post discharge was identified as a cost-effective practice to identify those socially isolated patients and implement necessary referrals early [34].

Establishing an adequate home environment is more critical in the current healthcare climate of moving care away from inpatient settings to more ambulatory and community settings [2]. Currently, there is very little literature to establish what a safe physical home environment might consist of. The participants in this current study have gone some way towards determining what a safe physical environment might be with the identification that patients need to enter and exit their house and be independent with functional chair, bed and toilet transfers and the resultant provision of equipment and home modifications to allow this. This current study recognized that all other home modifications could be completed once the patient is at home using community services. Given that this study identified the importance of a safe physical and robust social environment, health services and governments could assess funding options to ensure that there is adequate and appropriate funding for the provision of community and ambulatory services and care to facilitate these environments, shorten the length of stays and decrease hospital readmissions.

### Strengths and limitations

This study begins to address the gap in the literature by exploring the key factors that indicate a patient’s readiness for discharge from subacute care from the perspectives of multiple stakeholders. The included stakeholders in this study span across those with lived experience (inpatients and family members) to clinicians and managers. Currently, the majority of the literature exploring discharge-readiness is presented as separate stakeholder views, whereas this study presents a synthesis of a mixed population. There were several limitations to this study. Firstly, we excluded patients with cognitive deficits and those from non-English speaking backgrounds to ensure that all participants understood the project’s scope. These clinical populations may have unique discharge needs. We have attempted to reduce selection bias by including clinicians who treat non-English speaking patients and patients with cognitive deficits, and family members of these populations. Secondly, this study was conducted at a single site, which may limit the transferability of the results. Finally, the lead researcher was a senior clinician at the site which may have led to responder bias, however we minimized the impact of this through excluding patients under the lead researchers’ direct care.

### Implications for practice

Given the increased demand for healthcare services and the current move to shorter lengths of stays, healthcare services need to implement strategies to limit the risk of costly hospital readmissions. These strategies could include providing not only medications but accompanying this with an accurate medication list and providing education to the patient and/or family; providing equipment and assistance to manage continence and create a safe home environment while waiting for more permanent home modifications; and ensuring adequate utilization of services to manage and prevent readmissions for chronic health conditions. Education to patients and families need to facilitate the family’s familiarity of the patients changed abilities, potential safety risks and how to manage and reduce these as well as maximizing their potential for involvement in rehabilitation within the home setting, in the absence of clinicians to mitigate any wait times for community services.

## Conclusion

Discharging patients from hospitals is complicated. To date, there has been a lack of research on a range of key stakeholders’ perceptions of the factors of discharge-readiness in subacute care. This study concluded that environmental and personal characteristics are key factors for determining discharge-readiness.

## Data Availability

The datasets generated from this study are not publicly available due to reasons of confidentiality. Additional knowledge of the de-identified data is available from the corresponding author.

## References

[1] Bauer M, Fitzgerald L, Haesler E, Manfrin M (2009). Hospital discharge planning for frail older people and their family. Are we delivering best practice? A review of the evidence. J Clin Nurs.

[2] Auslander GK, Kaplan G, Ben-Shahar I, Soskolne V, Stanger V (2008). Discharge planning in acute care hospitals in Israel: Services planned and levels of implementation and adequacy. Health Soc Work.

[3] Shepperd S, Lannin NA, Clemson LM, McCluskey A, Cameron ID, Barras SL. Discharge planning from hospital to home. Cochrane Database Syst Rev. 2013(1):CD000313-CD. 10.1002/14651858.CD000313.pub4 23440778

[4] Robertson L, Blaga L (2013). Occupational therapy assessments used in acute physical care settings. Scand J Occup Ther.

[5] Heine J, Koch S, Goldie P (2004). Patients’ experiences of readiness for discharge following a total hip replacement. Aust J Physiother.

[6] Almborg A-H, Ulander K, Thulin A, Berg S (2009). Patients’ perceptions of their participation in discharge planning after acute stroke. J Clin Nurs.

[7] Lapum JPMNB, Angus JEPMB, Peter EPBAMB, Watt-Watson JPMB (2011). Patients’ discharge experiences: returning home after open-heart surgery. Heart Lung.

[8] Kozlowski D, Hutchinson M, Hurley J, Rowley J, Sutherland J (2017). The role of emotion in clinical decision making: an integrative literature review. BMC Med Educ.

[9] Longley V, Peters S, Swarbrick C, Bowen A (2019). What factors affect clinical decision-making about access to stroke rehabilitation? A systematic review. Clin Rehabil.

[10] Bucknall T (2003). The clinical landscape of critical care: nurses’ decision-making. J Adv Nurs.

[11] Poulos CJ, Magee C, Bashford G, Eagar K (2011). Determining level of care appropriateness in the patient journey from acute care to rehabilitation. BMC Health Serv Res.

[12] Gledhill K, Hanna L, Nicks R, Lannin NA. Defining discharge-readiness from subacute care from all stakeholders’ perspectives: a systematic review.Disabil Rehabil. 2020:1–8. 10.1080/09638288.2020.1733107 32126189

[13] Doyle L, McCabe C, Keogh B, Brady A, McCann M (2020). An overview of the qualitative descriptive design within nursing research. J Res Nurs.

[14] Saldaña J, Ebooks C (2011). Fundamentals of qualitative research.

[15] Hansen EC. Successful qualitative health research: a practical introduction. Crows nest, N.S.W.: crows nest, N.S.W. Allen & Unwin; 2006.

[16] Green J. Qualitative methods for health research. Thorogood N, editor. London Thousand Oaks, Calif: London Thousand Oaks, Calif: Sage Publications; 2004.

[17] Liamputtong P (2011). Focus group methodology: principle and practice.

[18] Phillippi J, Lauderdale J (2018). A guide to Field Notes for qualitative research: Context and Conversation. Qual Health Res.

[19] Braun V, Clarke V (2006). Using thematic analysis in psychology. Qualitative Res Psychol.

[20] Braun V, Clarke V (2021). One size fits all? What counts as quality practice in (reflexive) thematic analysis?. Qualitative Res Psychol.

[21] Fields NL (2016). Exploring the personal and environmental factors related to length of stay in assisted living. J Gerontol Soc Work.

[22] Myint PK, Vowler SL, Redmayne O, Fulcher RA (2008). Cognition, continence and transfer status at the time of discharge from an Acute Hospital setting and their Associations with an unfavourable discharge outcome after stroke. Gerontology.

[23] Kushner DS, Johnson-Greene D (2018). Association of urinary incontinence with cognition, transfers and discharge destination in Acute Stroke Inpatient Rehabilitation. J Stroke Cerebrovasc Dis.

[24] Talley KMC, Davis NJ, Peden-McAlpine C, Martin CL, Weinfurter EV, Wyman JF (2021). Navigating through incontinence: a qualitative systematic review and meta-aggregation of the experiences of family caregivers. Int J Nurs Stud.

[25] Dibley L, Coggrave M, McClurg D, Woodward S, Norton C (2017). It’s just horrible”: a qualitative study of patients’ and carers’ experiences of bowel dysfunction in multiple sclerosis. J Neurol.

[26] Basu J, Avila R, Ricciardi R (2016). Hospital Readmission Rates in U.S. States: are Readmissions higher where more patients with multiple chronic conditions cluster?. Health Serv Res.

[27] Patrik M, Lydia H, Tommy E, Anna B, Bengt L, Håkan W (2008). Medication report reduces number of medication errors when elderly patients are discharged from hospital. Pharm World Sci.

[28] Ziaeian B, Araujo KLB, Van Ness PH, Horwitz LI (2012). Medication Reconciliation Accuracy and Patient understanding of intended medication changes on Hospital Discharge. J Gen Intern Med.

[29] Tong EY, Roman CP, Mitra B, Yip GS, Gibbs H, Newnham HH (2017). Reducing medication errors in hospital discharge summaries: a randomised controlled trial. Med J Aust.

[30] Parekh N, Ali K, Stevenson JM, Davies JG, Schiff R, Van der Cammen T (2018). Incidence and cost of medication harm in older adults following hospital discharge: a multicentre prospective study in the UK. Br J Clin Pharmacol.

[31] Nguyen T, Nguyen TH, Nguyen PT, Tran HT, Nguyen NV, Nguyen HQ (2018). Pharmacist-led intervention to enhance medication adherence in patients with Acute Coronary Syndrome in Vietnam: a Randomized Controlled Trial. Front Pharmacol.

[32] Dreyer K, Steventon A, Fisher R, Deeny SR (2018). The association between living alone and health care utilisation in older adults: a retrospective cohort study of electronic health records from a London general practice. BMC Geriatr.

[33] Ha J-H, Hougham GW, Meltzer DO (2019). Risk of social isolation among older patients: what factors affect the availability of family, friends, and neighbors upon hospitalization?. Clin Gerontol.

[34] Lewis E, Samperi S, Boyd-Skinner C (2017). Telephone follow-up calls for older patients after hospital discharge. Age Ageing.

